# Lipoma of vallecula—a case report

**DOI:** 10.1259/bjrcr.20150460

**Published:** 2016-07-28

**Authors:** Kia Sing Tan, Win Mar Salmah Jalaluddin

**Affiliations:** Department of Radiology, Hospital Universiti Sains Malaysia, Kubang Kerian, Malaysia

## Abstract

Lipomas of the larynx, oropharynx and hypopharynx are rare, accounting for approximately 1% of benign laryngeal neoplasms. We present a rare case of a simple lipoma arising from the right vallecula. A 55-year-old male presented with worsening dysphagia for 1 week. CT scan revealed a lesion of fat attenuation in the right vallecula. The patient underwent surgical excision and recovered uneventfully. To our knowledge, there are only two cases of vallecular lipoma that have been reported and they are both of spindle cell subtype and located on the left side. This is the first reported case of a simple lipoma arising from the right vallecula and causing obstructive symptoms. CT scan or MRI is essential in confirming the diagnosis and assessing the extent, thus allowing prompt excision, especially when the patient is symptomatic.

## Background

Lipomas are one of the most common benign neoplasms found in humans, but occurrence in the oropharynx is rare. Their overall incidence in the oral cavity and oropharynx is between 1% and 4.4% of all benign oral lesions. The more common anatomical sites for oral lipomas are the major salivary glands, buccal mucosa, lip, tongue, palate, vestibule and floor of the mouth.^1^ We present a case of a simple lipoma arising from the right vallecula. To the best of our knowledge, only two cases of vallecular lipomas have been described, both of which were spindle cell variants and located on the left side.^2,3^

## Clinical presentation

A 55-year-old Malay male presented to our hospital with a history of dysphagia for 1 year, worsening over the past 1 week. He was only able to tolerate soft diet and liquids. Occasionally, he experienced odynophagia and mild shortness of breath. However, there was no stridor and hoarseness of voice. He did not complain of neck swelling or any ear symptoms. On examination, there was a smooth, well-defined, non-lobulated soft tissue mass between the base of the tongue and epiglottis. It occupied about 70–80% of the oropharyngeal opening. Endoscopy showed no extension of the mass into the laryngeal inlet. Both vocal cords were mobile and symmetrical. The aryepiglottic folds and pyriform sinuses revealed no abnormality.

## Imaging findings

**Figure 1.**
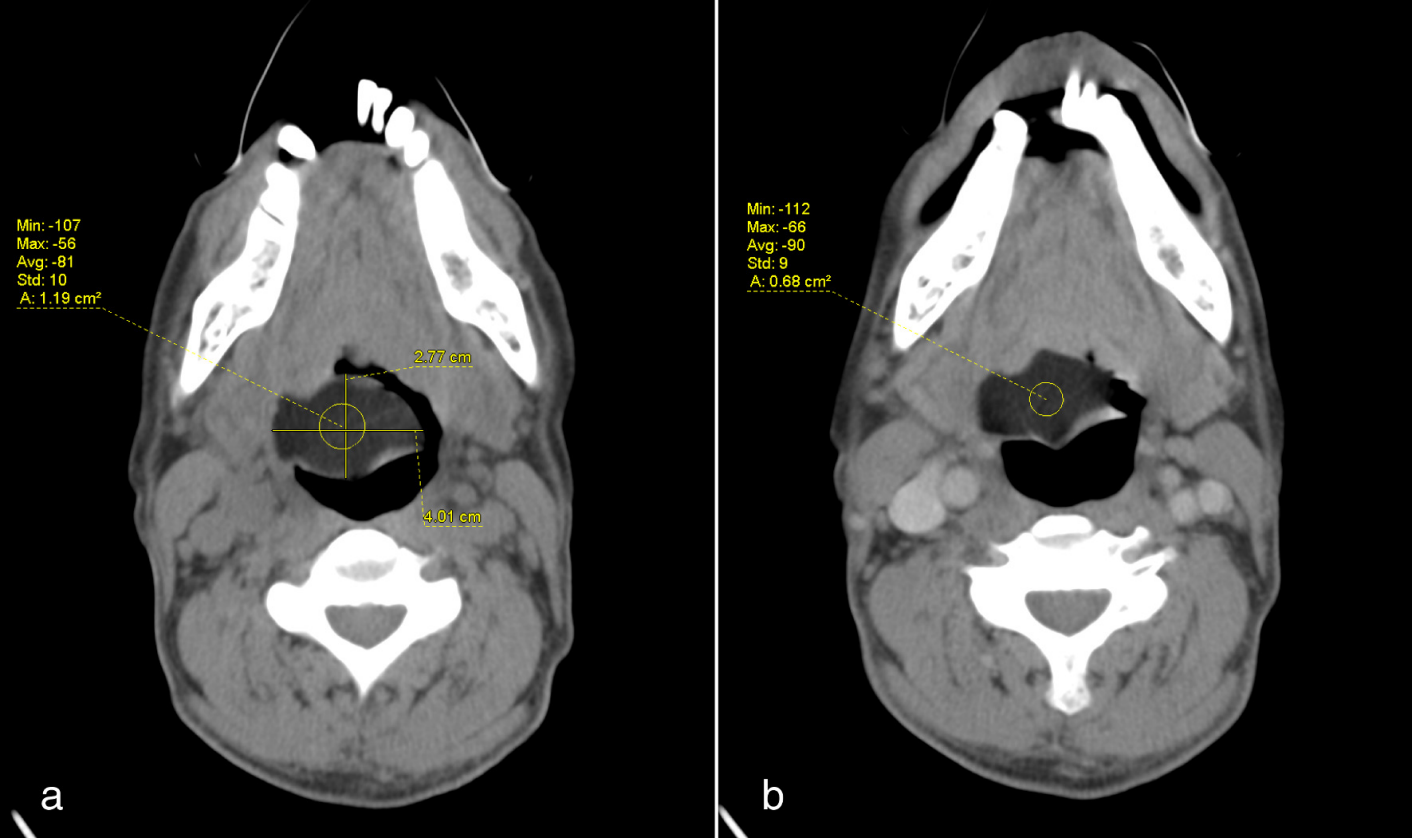
(a) Plain and (b) contrast-enhanced CT scan showing a hypodense lesion at the right vallecula with mean Hounsfield unit of −81, suggestive of fat content. No significant enhancement is seen post contrast.

**Figure 2.**
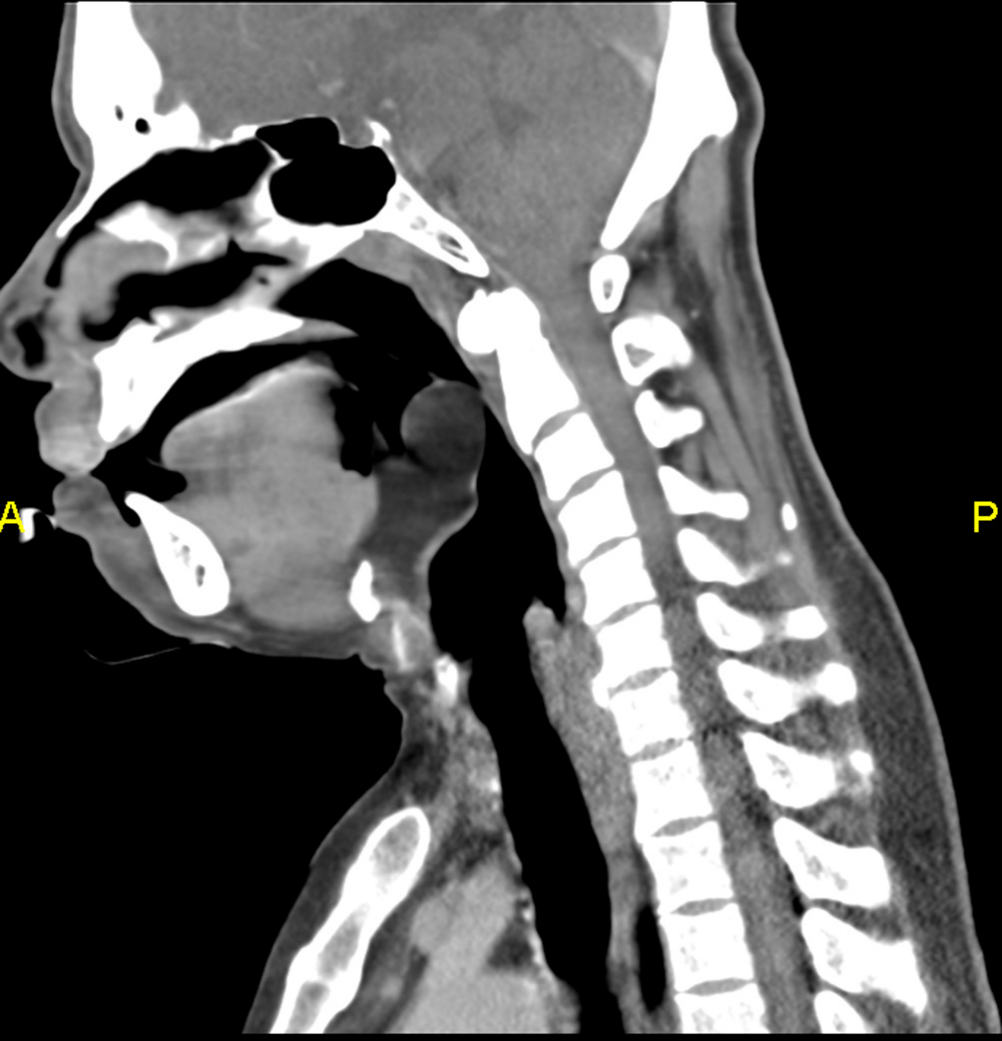
The same lesion as it appears on sagittal reformatted image. It is broad-based, attached to the lingual surface of the epiglottis and free from the base of the tongue.

**Figure 3.**
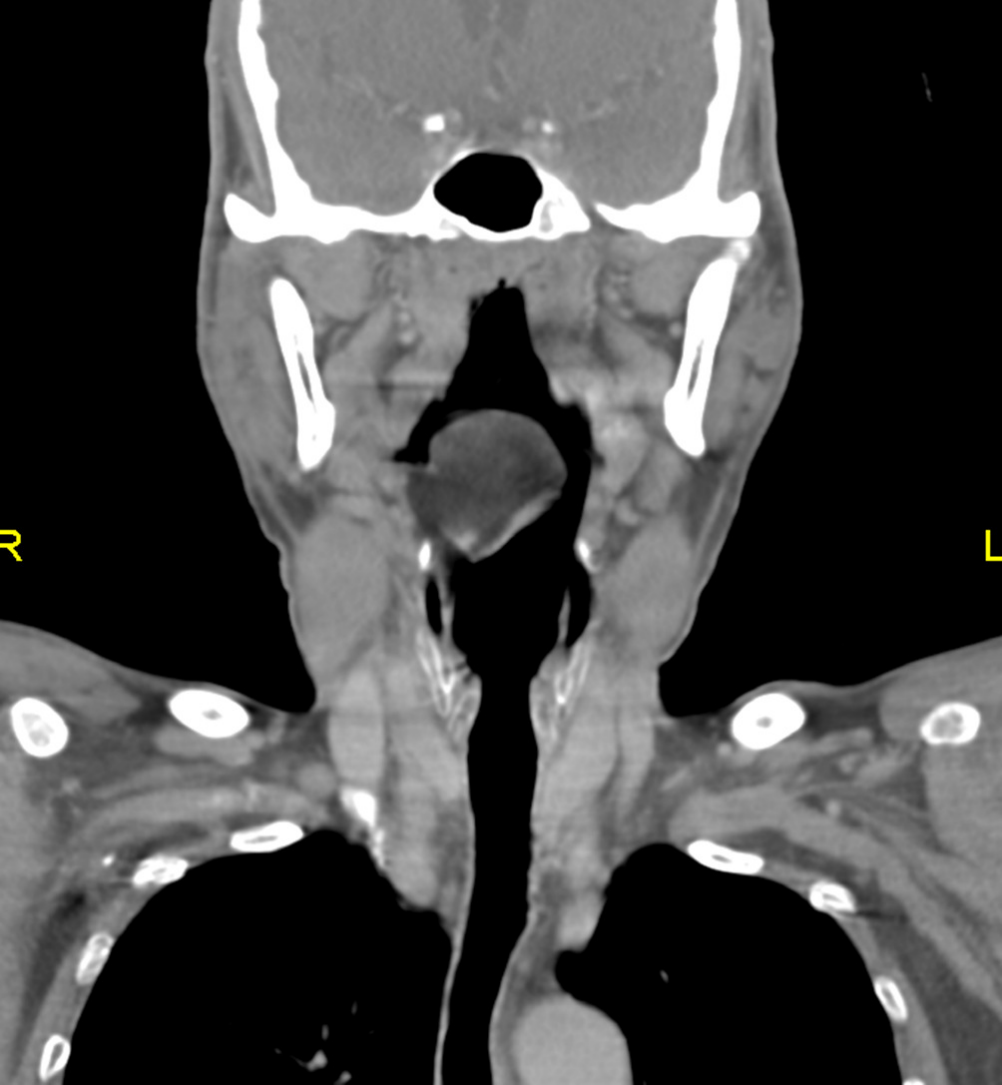
The same lesion on coronal reformatted image showing narrowing of the oropharynx. The hypopharynx is still spacious.

CT scan of the neck showed a well-defined, smooth margin, hypodense lesion occupying the right vallecula and measuring 2.8 × 4.0 cm in size. It had a mean attenuation of −81 HU (Figure 1). There was no significant enhancement after contrast administration. It was broad–based. attached to the lingual surface of the epiglottis and had a plane of demarcation with the base of tongue, which was best seen on the sagittal reformatted image (Figure 2). The oropharynx was narrowed but not obstructed. The larynx and hypopharynx were normal (Figure 3). A diagnosis of right vallecular lipoma was made.

## Treatment and follow-up

The patient underwent endoscopic excision of the lipoma under general anaesthesia. Intraoperatively, the mass was seen to partially occupy the lingual surface of the epiglottis and the right vallecula. It was excised into without any complications. Histopathological diagnosis was of right vallecular lipoma. The lesion was composed of mature univacuolated adipocytes separated by thin fibrovascular septae and covered with non-keratinizing squamous epithelium. Postoperatively, the patient recovered well, with resolution of symptoms. Follow-up endoscopy at 1 month showed no recurrence.

## Discussion

Lipomas are the most common benign mesenchymal tumours that arise from mature white fat cells. They occur everywhere in the body. However, only about 15–20% of them occur in the head and neck region and 1–4% in the oral cavity.^4^ Oral lipomas with decreasing frequency occur in the cheek, tongue, floor of the mouth, buccal sulcus, palate, lip and gingiva.^5^

Lipomas are usually found in patients after 30 years of age, although they may also be congenital. They may occur at any time between early childhood and advanced age, with more than 50% being found in the fifth to sixth decades, when fat begins to accumulate in inactive, underexercised individuals. Occurance is twice as high in females as in males.^6^

Even though they are benign lesions, they can be life-threatening owing to their location. Morphologically, oral lipomas may be sessile or pedunculated, and thus can present with different symptoms. A few cases of pedunculated lipoma at the epiglottis, posterior cricoid and aryepiglottic fold causing laryngeal obstruction leading to death have been reported.^7^ Otherwise, the sessile lipomastend to be asymptomatic but may cause obstructive symptoms such as dysphagia and stridor when their size increases.^7^ Our patient had dysphagia for a year, indicating that the lesion was slow-growing. Later, it was large enough to occupy 70–80% of the oropharyngeal space.

CT scan has the ability to definitively diagnose a lipoma in virtually all cases owing to its typical characteristic feature—homogeneous low attenuation that measures between −65 and −125 HU. They are non-enhancing. No clear identifiable capsule can be seen, but most lesions are easily delineated from the adjacent soft tissues. The other possible diagnosis could be a ranula or dermoid, which have characteristics similar to lipoma.^5^ MRI is superior than CT in evaluation of soft tissue and tumour extension; it is, however, not readily accessible and is more time consuming.^2^ Our patient definitely showed the typical findings of lipoma on CT imaging.

Histologically, lipomas can be classified as either simple ordinary lipomas or their variants, which include spindle cell lipomas, fibrolipomas, intramuscular lipomas, angiolipomas, myxolipomas, salivary gland lipomas, pleomorphic lipomas and atypical lipomas.^8^ Simple lipomas make up 80% of the oral lipomas while all the other variants constitute the remaining 20%. Our patient had the simple lipoma histology, but at a rare location.

The recommended treatment for pharyngeal and laryngeal lipomas is surgical excision; the extent of the procedure depends on the size and site of the tumour. In our case, surgery was indicated as the lipoma was obstructing the oropharynx and causing dysphagia. It had the potential to cause a life-threatening event by obstructing the airway. Endoscopic removal was recommended but large masses would need an external approach. Complete removal is essential to reduce the risk of recurrence.^9^ The prognosis for this disease is excellent. However, long-term follow-up is needed owing to the possibility of initial missed diagnosis of liposarcoma as histological differentiation of lipoma and low-grade liposarcoma can be difficult.^2,3^

## Learning points

1. Even though lipomas are benign tumours and slow-growing, they can cause life-threatening conditions if they occur at certain locations, such as in the oropharynx and larynx.
2. CT scan or MRI is important in demonstrating the fat content to confirm the diagnosis of vallecular lipoma and helpful in assessing the extent prior to surgical removal.
3. Long-term follow-up is needed as there is a possibility of initial missed diagnosis of liposarcoma.

## Consent

Written informed consent was obtained from the patient for publication of this case report, including accompanying images. The record is held by the corresponding author.
